# Effects of cold storage on quality of *Chrysopa pallens* and recovery of fecundity by insulin

**DOI:** 10.1038/s41598-019-41618-y

**Published:** 2019-03-29

**Authors:** Tingting Zhang, Guocai Zhang, Lisheng Zhang, Hongyin Chen, Mengqing Wang, Chenxi Liu, Jianjun Mao

**Affiliations:** 1 School of Forestry, Northeast Forestry University, Harbin, Heilongjiang 150040 China; 2 Key Laboratory for Biology of Plant Diseases and Insect Pests, Ministry of Agriculture, Institute of Plant Protection, Chinese Academy of Agricultural Sciences, Beijing, 100193 China

## Abstract

The green lacewing, *Chrysopa pallens* Wesmael, is one of the most beneficial and prolific insects found in many horticultural and agricultural cropping system. Here, the effects of low temperature storage on quality of *C. pallens* were investigated by storing cocoons at 10 °C for different days. Results revealed, after removal from cold storage, emergence rate declined gradually as storage duration increased. After storage of 20 days, the emergence rate in cold-stored group is about 62.8% of that in unstored group. After eclosion, lifetime fecundity, preemergence period, oviposition period and longevity of adults in cold-stored group showed curves similar to emergence rate. However, preoviposition period and egg hatchability were not significantly affected by cold. After being stored for 20 days, the total fecundity of females emerging from cold-stored cocoons was about 64.5% of that of females emerging from unstored cocoons. Six days post emergence, females in cold-stored group showed apparent arrest of ovarian development and significant reductions of protease, lipase and trehalase activities when compared to unstored controls. When bovine insulin was exogenously used, the females emerging from cold-stored cocoons dramatically restored ovarian development and reproductive capacity. These results suggested that *C. pallens* pupae are suitable for cold storage and insulin hormone can be used as reproduction stimuli in this predatory species after cold storage.

## Introduction

The green lacewing, *Chrysopa pallens* Wesmael, is one of the most effective natural predators and has distribution in most agricultural areas of the world^1,2^. The larval green lacewings are commonly known as “aphid lions” and prey most soft-bodied insects of suitable size, including aphids, caterpillars, and other insect larvae and eggs^2,3^. *C. pallens* is different from other green lacewings in that both its adult and larval are predators^1,4,5^. *C. pallens* females possess robust fecundity, laying about 1000 eggs in an oviposition period under controlled conditions^6,7^. Therefore, *C. pallens* is an excellent predator and has very attractive application potential in management of insect pests.

In recent decades, there has been increasing interest in mass rearing and commercial application of natural enemies, including parasitoids and predators^2,8–10^. One of the main obstacles in efficient biological control using large-scale release is the failure to obtain large enough number of natural enemies during periods of high demand^11,12^. When pest population changes with the season, natural enemies may be overproduced and underproduced because of fluctuation in demands^13,14^. So, it is crucial to develop storage techniques for natural enemies because prolonged shelf-life improves flexibility, reliability and efficiency in commercial production^8,14–17^. Stinner^18^ considered storage techniques were critical to the reduction of cost. Many studies revealed that most parasitoids can endure short-period cold storage with minimal impairment in fitness and viability^19–24^. In these studies, important biological characteristics, including survival, longevity, fecundity and behavior, were evaluated^17,25–29^. Among the different developmental stages, pupal stage is considered to be most suitable for short-term cold storage because pupae have stronger cold tolerance than eggs, larvae and adults^30^.

Taken together, cold storage of parasitoids has received considerable interest. However, that of predators like green lacewing did not. The larvae of *C. pallens* feed for 2–3 weeks before pupation. The pupal stage lasts for about 10 days and then emergence take place^2,4,31,32^. If *C. pallens* is subjected to cold storage at pupae stage, the poststorage adults will be brought into a reproductive sate rapidly and predictably. So, it is very meaningful to evaluate the effects of cold storage on this species.

Now, cold storage has been accepted as a very practical method for improving production efficiency of natural enemies, but the detrimental effects of cold storage, such as decline of survival and fecundity, have also been extensively recognized^17,22,33^. Therefore, how to minimize the adverse effects is an inevitable problem and should be soon taken into consideration. Recently, insulin like peptides (ILPs) was found playing crucial roles in reproductive regulation of the green lacewing. Application of bovine insulin promoted ovarian development and elevated reproductive output in lacewing female adults kept at rearing conditions^34^, implying the application prospect of insulin hormone in production of natural enemies insects.

In this paper, *C. pallens* pupae were maintained at 10 °C for different days and then transferred to rearing conditions. After removal from cold storage, most biological parameters such as emergence rate, lifetime fecundity, preemergence period, oviposition period and longevity of the emerged adults declined gradually with the increase of storage duration. Ovarian development was arrested and digestive enzyme activities were reduced by cold storage. But fortunately, the emergence and reproduction of females emerging from cold-stored cocoons was not seriously damaged after being stored for 20 days. After application of bovine insulin, ovarian scale and reproductive output were dramatically restored. These results suggested that cold storage was a useful technique for improving flexibility and efficiency in mass production of *C. pallens* and insulin hormone could be applied as reproduction stimuli after chilling treatment.

## Materials and Methods

### Insect rearing

The *C. pallens* colony was fed with pea aphid (*Acyrthosiphon pisum*) and kept at 25 °C and 70% relative humidity (RH) under a 16 h light: 8 h dark photoperiod (D). After eclosion, males and females were reared together for mating.

### Cold storage treatment and its effects on biological parameters

For cold storage, 10 cocoons (24-h old) were contained in a plastic box with a length of 15 cm, width of 20 cm and height of 7.5 cm and kept at 10 °C, 70% RH in darkness for 10, 20, 30, 40, 50 and 60 days. At least 6 boxes were randomly selected for each storage treatment. After storage of different period, the cocoons were transferred to rearing conditions (25 °C, 70% RH, 16 h light: 8 h dark) for emergence. Sixty cocoons (24-h old) consistently maintained at normal rearing conditions were served as control. Following parameters were evaluated: emergence rate (number of emerged adults/number of cocoons); preemergence period after removal of cocoons from cold storage, preoviposition period; oviposition period; female longevity; total fecundity and egg hatching. For hatchability, 50–100 eggs laid by females of the same storage treatment were randomly selected every time on day 12–18th post emergence. Neonates were timely transferred and recorded. The hatching assay was repeated 3–5 times for each storage treatment.

### Effects of cold storage on enzyme activity in female adult

After 20 days cold storage, digestive enzyme activity in emerged female adults (5-day old) was examined. The trehalase activity was measured using insect trehalase ELISA kit (SU-B97117, Collodi, Quanzhou, China) according to manufacturer’s manual. Five days after eclosion, female adults were weighted and ground individually in liquid nitrogen. Every 100 mg insect tissue was suspended by 1 ml PBS buffer. The mixture was centrifuged at 10000 g for 20 min at 4 °C. The supernatant (50 μl) and standards (50 μl) were added in duplicate to wells of a microtiter plate. Enzymeconjugate (100 μl) was added to each standard and sample well. The plate was covered with an adhesive strip and incubated at 37 °C for 1 h. The wells were briefly rinsed with washing buffer for 4 times. Substrate A (50 μl) and substrate B (50 μl) were added to each well to develop color. After a dark incubation at 37 °C for 15 minutes, stop solution (50 μl) was added to each well to terminate reaction. The immunoassay plate was analyzed for absorbance with FlexStation 3 (Molecular Devices, California, USA) at 450 nm within 15 minutes.

The protease activity was detected using Pro ELISA kit (SU-B97122, Collodi, Quanzhou, China), and lipase activity was monitored by Lipase ELISA kit (SU-B97006, Collodi, Quanzhou, China) according to procedures described in trehalase activity measurement.

### Effects of cold storage on ovarian development

After storage for 20 days, the emerged females (5-day old) were dissected in saline solution and ovaries were microscopically observed at 30-fold magnifications (VHX-2000, Keyence, USA). Three ovarioles were randomly selected from a single ovary and lengths of primary follicles (1 fol) and secondary (2 fol) follicles were measured.

### Preparation and injection of bovine insulin

Bovine insulin (Nacalai, Kyoto, Japan) and Bull Serum Albumin (BSA) were solubilized in 25 mM HEPES (pH 8.2) at 10 mg/ml^34^. After cold storage for 20 days, the emerged females was injected 1 μl insulin solution at the conjunctive between the fourth and fifth abdominal segment on day 2 after emergence. Injection was performed in 0.2 s under a pressure of 400 mP using Eppendorf InjectMan® NI 2 and FemtoJet express Microinjector (Eppendorf, Hamburg, Germany). Females emerging from cold-stored cocoons were injected the same volume of BSA solution and were served as controls. At least 30 females were injected in each treatment. Egg amount was recorded daily after injection.

### Real-time PCR

To detect mRNA levels of *C. pallens ILP1* (*CpILP1*) (GenBank accession number:MH753707), *C. pallens ILP2* (*CpILP2*) (GenBank accession number:MH753709), *C. pallens ILP3* (*CpILP3*) (GenBank accession number: MH753708) in females from cocoons cold-stored for 20 days, total RNA was extracted 3days after emergence using TransZol Up Plus RNA Kit (Transgene, Beijing, China). Elimination of genomic DNA contamination and synthesis of first-strand cDNA were realized simultaneously by using TransScript® One-Step gDNA Removal and cDNA Synthesis SuperMix (Transgene, Beijing, China). Quantitative PCR was performed in a 20 μl reaction volume containing 200 nM each of forward and reverse primers, 8 μl nuclease-free water, 10 μl 2 × iTaq universal SYBR Green supermix (BIO-RAD, California, American), and cDNA produced from 2 μg total RNA. The PCR reaction was carried out by a 7500 real-time system (Applied Biosystems, California, USA). The housekeeping gene actin^35^ (GenBank accession number: KC505163.1) and ribosomal protein encoding gene S26e^36^ (GenBank accession number: MH753710) was used as internal control for normalization. Sequences of gene-specific qRT-PCR primer sets are listed in Table 1. Individual animals were randomized into 2 treatment groups, and 3 replications were performed for each treatment. Quantification of relative changes in gene transcripts abundance was performed according to the 2^−ΔΔCt^ method^37^.

**Table 1:** Primers used in quantitative PCR.

Gene	Forward (5′-3′)	Reverse (5′-3′)	Fragment size (bp)
*CpILP1*	TGTATGTCAAGGATTCACTAATG	AACAATACCAGTCCGCTTA	193
*CpILP2*	AAACCAATCGTCACAAAC	GGGGTGGAATATCATCTC	119
*CpILP3*	TACAATGGGCATGTGATG	GTGTACGAAATGGAAACG	135
*actin*	TCCAGAAGAACACCCAATCC	ATACACCATCACCAGAGTCAAGT	188
ribosomal protein *S26e*	CGAAACAGAAGTAAAACCGAAC	TGCGTTGTTGTTGTGGAT	91

## Results

### Effects of cold storage on emergence, reproduction and longevity of *C. pallens*

**Figure 1:**
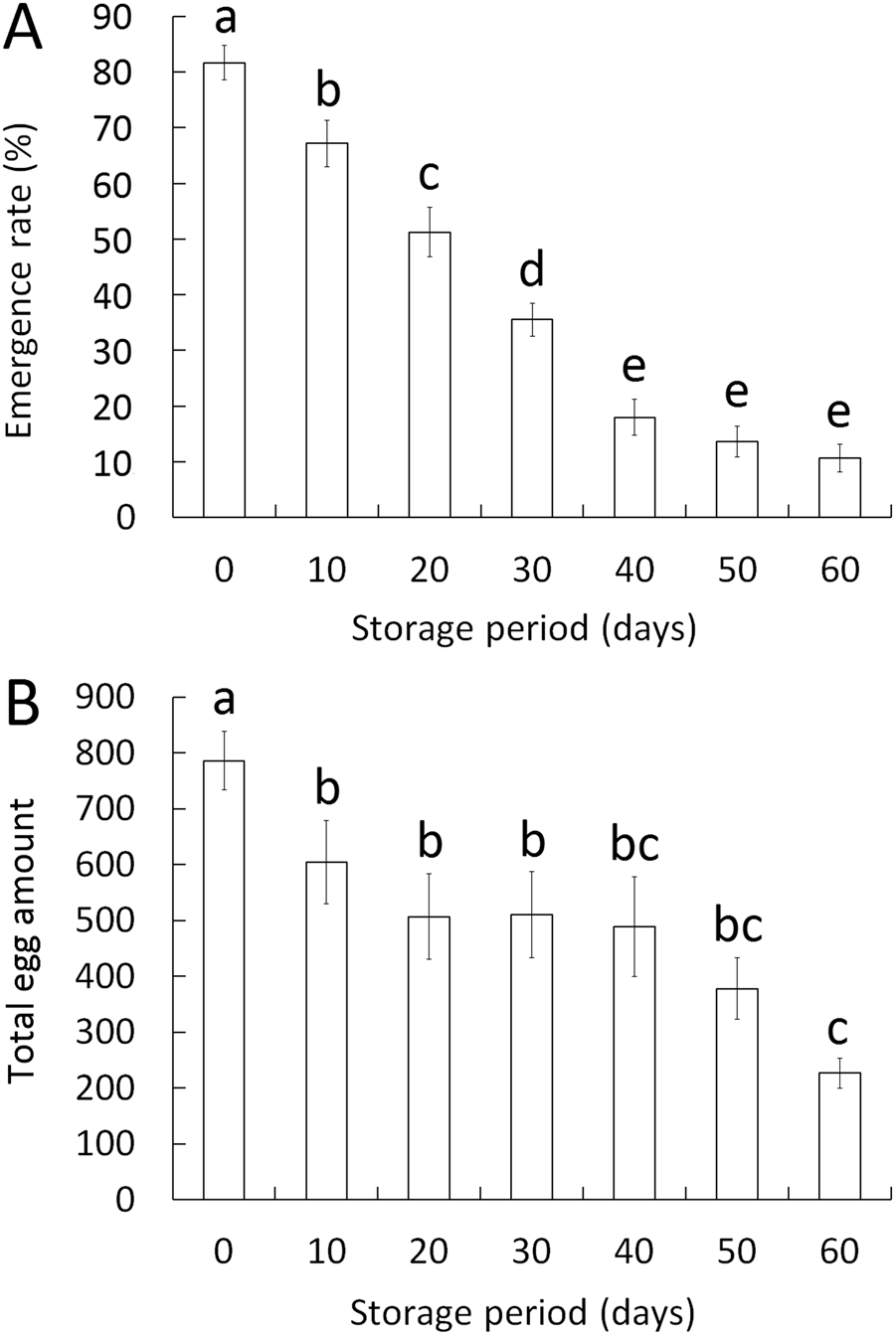
Effects of cold storage on emergence rate and lifetime fecundity of *C. pallens*. (**A**) Emergence rate of *C. pallens* after cold storage. The emergence of *C. pallens* after removal from cold storage decreased gradually as the storage duration extended (n = 6–15). (**B**) Total fecundity after cold storage. Total egg amount per female was significantly lower in cold storage group than that in control group (n = 6–23). Bars (mean ± SE) denoted by the same letter are not significantly different (ANOVA with LSD, *P* < 0.05).

The emergence of *C. pallens* after removal from cold storage declined gradually as the storage period elongated. After storage of 20 days, about 51.3% cocoon emerged and the difference between the cold storage group and the control group was significant. The emergence was only about 10.7% when low temperature was kept for 60 days (Fig. 1A).

With the extension of cold storage duration, reproduction of female adults also went down gradually. Lifetime fecundity of females emerging from cocoons cold-stored for 10 and 20 days dropped by about 23.1% and 35.5%, respectively, when compared to that of control group. After storage of 40 days, lifetime fecundity decreased further but was still 62.1% of that in unstored group. When storage lasted for two months, reproduction was seriously damaged with a total egg amount of 226.5 per female (Fig. 1B).

Similarly, the preemergence period also dropped with the increase of cold storage period. After storage for 30 days, lacewings emerged significantly earlier than those from unstored controls. When cocoons were cold stored for 60 days, the preemergence period of adults was 4.8 days and less than half of that in control group (Table 2). The preoviposition period of the emerged adults was not markedly affected by cold storage and spanned from 5.9 to 6.7 days after different storage durations (Table 2). The oviposition period between the cold-stored and unstored groups did not vary significantly until cold storage continued for 50 days. When low temperature was kept for two months, oviposition period of emerged females was about 12 days shorter than that of unstored controls (Table 2). Similar to oviposition period, longevity of emerged adults showed no significant difference between the cold storage group and the control group until storage duration was increased to 50 days (Table 2). Egg hatching was not affected by cold storage. After storage for two months, hatchability of eggs oviposited by females emerging from cold stored cocoons still remained at a high level of 82.4% (Table 2).

**Table 2:** Reproductive parameters of *C. pallens* after cold storage.

Parameters	0d	10d	20d	30d	40d	50d	60d
Preoviposition period (days)	6.43 ± 0.41a	6.67 ± 0.33a	6.56 ± 0.60a	6.21 ± 0.52a	5.9 ± 0.62a	6.7 ± 0.43a	6.33 ± 0.74a
Oviposition period (days)	24.96 ± 2.39a	24.33 ± 1.58a	23.44 ± 2.02a	23.36 ± 2.31ab	22.00 ± 1.61ab	16.60 ± 3.02bc	12.67 ± 2.95c
Longevity (days)	31.82 ± 2.97a	30.45 ± 1.97a	29.44 ± 2.05a	30.21 ± 2.34a	28 ± 2.12a	22.5 ± 2.15b	18.11 ± 4.30b
Preemergence period (days)	10.18 ± 0.37a	9.95 ± 0.46a	9.50 ± 0.45a	7.29 ± 0.69b	6.90 ± 0.64bc	5.33 ± 0.53 cd	4.83 ± 0.60d
Hatching (%)	81.20 ± 1.71a	79.40 ± 3.12a	78.40 ± 3.01a	78.60 ± 2.50a	84.00 ± 2.66a	78.60 ± 2.79a	82.40 ± 1.69a

### Arrest of ovarian development after cold storage

**Figure 2:**
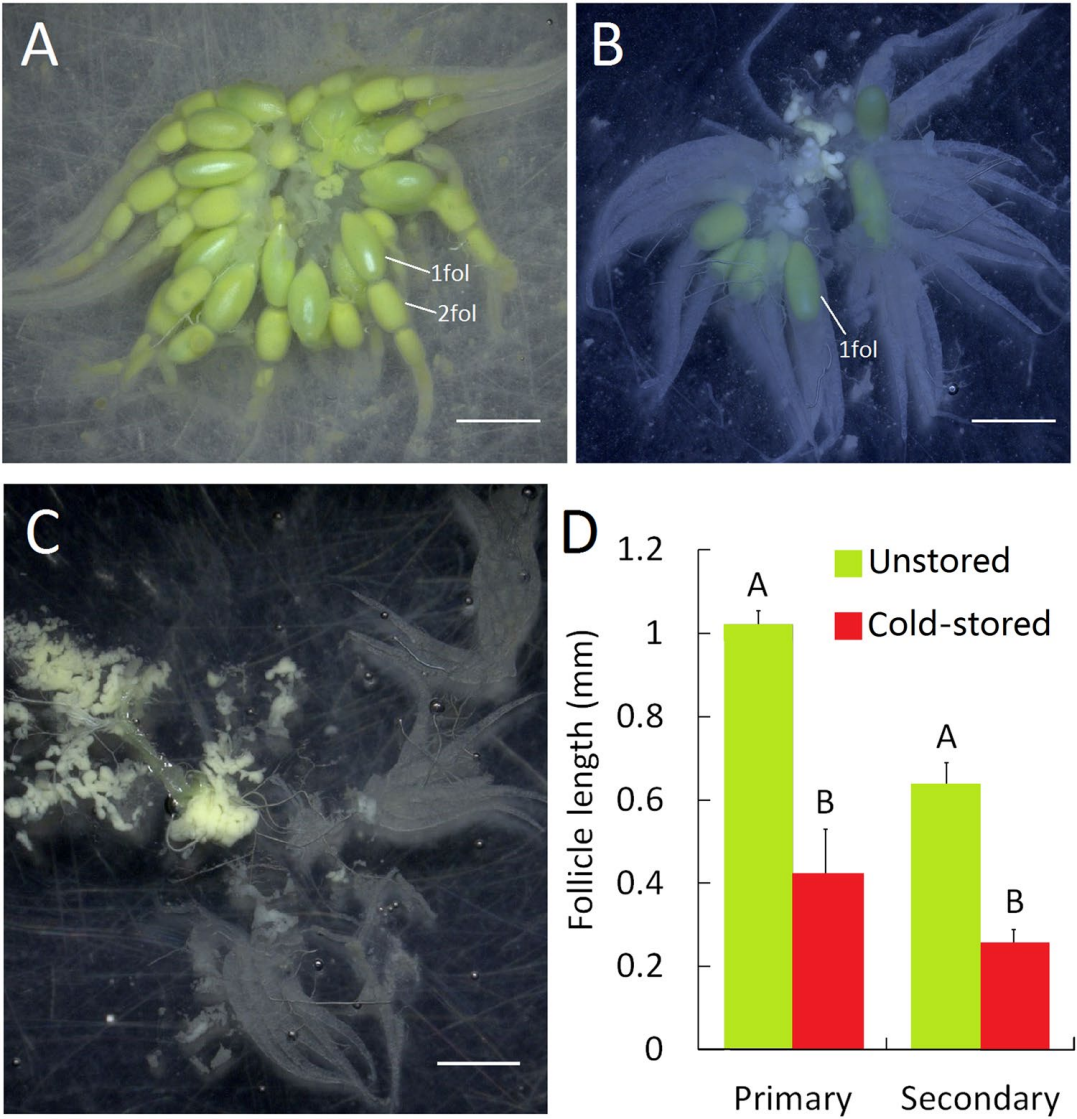
Ovarian development in *C. pallens* after cold storage. (**A**) Ovaries in control female without low temperature treatment. (**B**) Ordinary type of unmatured ovaries in females after cold storage showed an uneven development of the ovarioles. Only part of the ovarioles produced follicles of normal size. (**C**) Extreme type of unmatured ovaries in females after cold storage was still in an initial development state. The ovarioles was apparently smaller and yielded no matured follicles. (**D**) The primary follicles (1 fol) and secondary follicles (2 fol) in females after cold storage are significantly smaller in length than that in control females. Scale bar is 1 mm. Different letters above the bars indicate a significant difference (Student’s *t* test, *P* < 0.01), n = 9.

Female adults emerged from cold-stored cocoons were dissected 5 days post eclosion. In females emerged from unstored cocoons, all primary follicles (1 fol) were matured and the secondary follicles (2 fol) have formed (Fig. 2A). However, females from cold storage group showedless matured ovaries with smaller developmental scale and follicle size when compared to control group. In ordinary type of the unmatured ovaries in females emerging from cold-stored cocoons, the ovarioles showed uneven development. A minority of the ovarioles yielded matured primary follicles, but for most others, their primary follicles were obviously arrested (Fig. 2B). In extreme type of the unmatured ovaries in females emerging from cold-stored cocoons, the ovarioles was still in an initial development state and produced no visible follicles (Fig. 2C). The primary and secondary follicles in females from cold-stored cocoons were significantly smaller in size than those in females from control group (Fig. 2D).

### Reduction of digestive enzymes activities after cold storage

**Figure 3:**
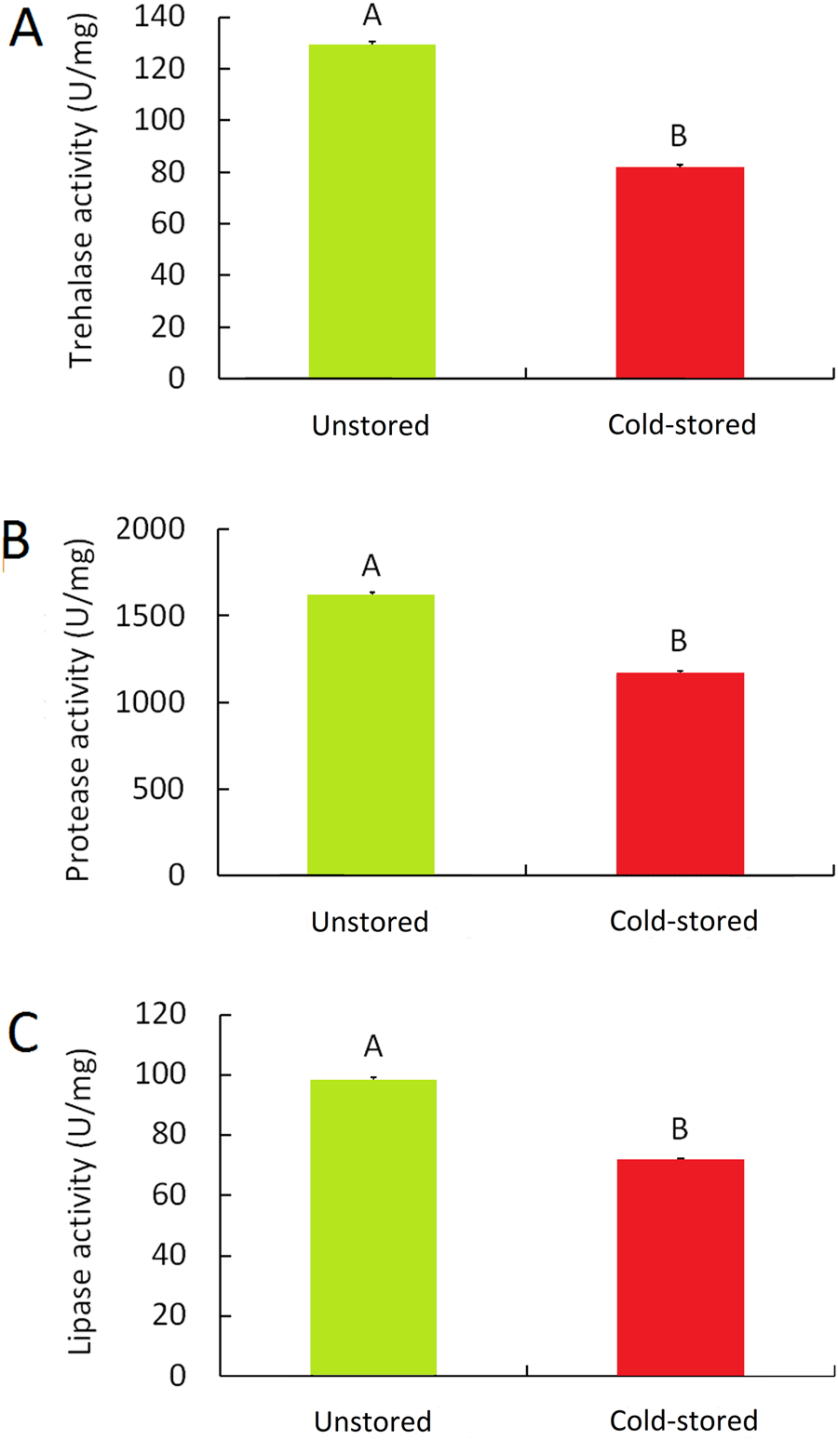
Digestive enzymes activities in females after cold storage. Five days post emergence, activity of trehalase (**A**) in females after cold storage was statistically weaker than that in control ones. Significant differences in protease (**B**) and lipase (**C**) activities were also observed between the cold storage group and control group. Mean ± SE (n = 3) of each bar followed by the same letter are not statistically different (Student’s *t* test, *P* < 0.01).

Five days post emergence, activity of trehalase in females was statistically lower in cold-stored group than in unstored group (Fig. 3A). Activities of protease (Fig. 3B) and lipase (Fig. 3C) showed a pattern consistent to that of trehalase with a significant difference between the cold-stored and the unstored groups.

### Resumption of ovarian development after insulin treatment

**Figure 4:**
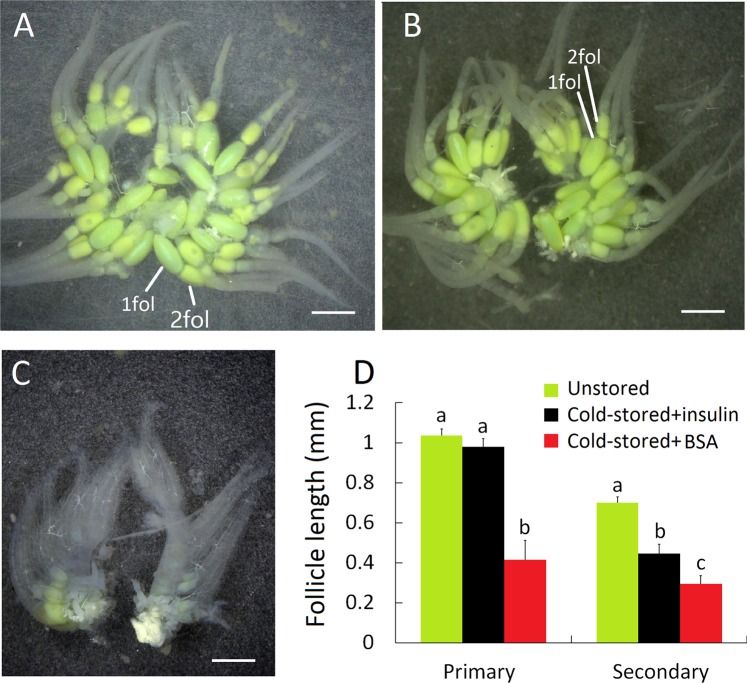
Resumption of ovarian development after injection of bovine insulin. (**A**) Ovaries in wild-type females. (**B**) Ovaries in cold storage females injected with insulin recovered development and showed uniform ovarioles with matured follicles comparable to the counter parts in wild-type females. (**C**) Ovaries in cold storage females treated with BSA remained unmatured. (**D**) Primary follicle (1 fol) and secondary follicle (2 fol) length in cold storage females increased dramatically after treatment of insulin hormone. Scale bar is 1 mm. Mean ± SE with the same letters are significantly different at *P* = 0.05, n = 9, ANOVA.

As demonstrated above, cold storage of cocoons inhibited ovarian development in female adults. However, topical application of bovine insulin rescued the development arrest. Five days post emergence, ovaries in control group showed normal scale and yielded matured follicles (Fig. 4A). Ovaries in insulin-treated females (Fig. 4B) restored development and their ovarioles formed matured primary follicles comparable to that in BSA-treated group. However, ovaries in females injected with BSA failed to recover development and remained unmatured (Fig. 4C). Primary and secondary follicle length in females emerging from cold-stored cocoons increased apparently after treatment of insulin hormone. Significant difference in follicle size was still present between the BSA treated group and the wild-type group, but absent between the hormone applied group and the wild-type group (Fig. 4D).

### Recovery of fecundity after application of insulin

**Figure 5:**
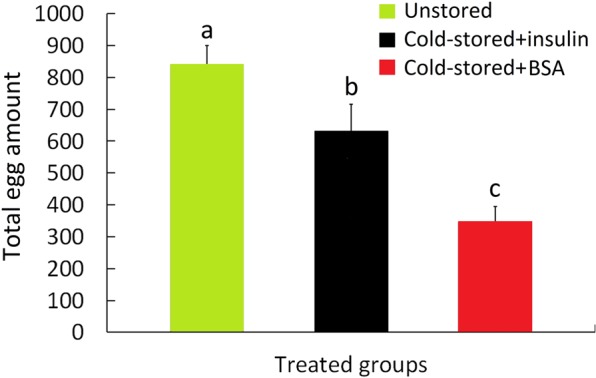
Recovery of fecundity after application of bovine insulin. Hormone treated females produced significantly more eggs that BSA treated ones. But significant difference in total fecundity (n = 30–40) was also observed between the hormone treated females and wild-type animals. Scale bar is 1 mm. Bars (Mean ± SE) with the same letters are significantly different at *P* = 0.05, ANOVA.

Because of recovery of ovarian development in females emerging from cold stored cocoons, bovine insulin treated females laid significantly more eggs than BSA treated individuals. However, significant difference in total fecundity was still present between the insulin treated females and the wild-type animals (Fig. 5).

### Decrease of *CpILP*s transcripts after cold storage

**Figure 6:**
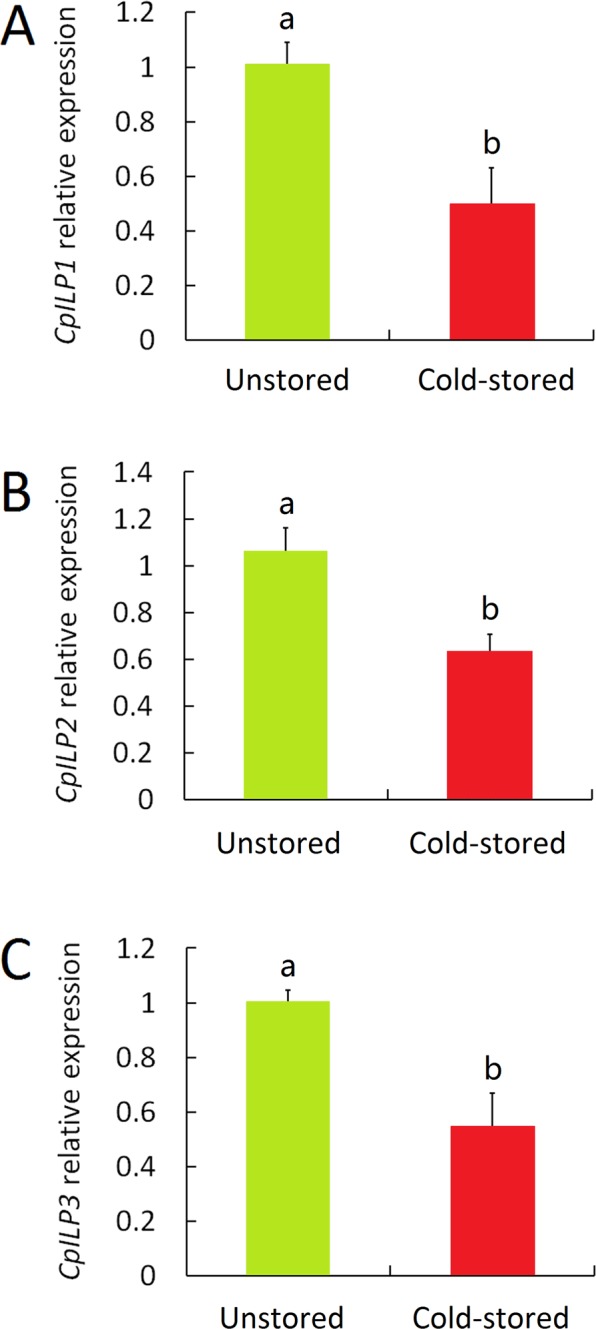
Relative expression of *C. pallens ILP*s in cold-stored lacewing. The mRNA levels of three *ILP*s were analyzed by qPCR. The relative expression of *ILP*s was normalized to the expression level of *actin*. Bars are Mean ± SE from three replicates. Bars with the same letters are significantly different at *P* = 0.05, Student’s *t* test.

The relative expression of the *C. pallens ILP*s revealed by using *actin* and *S26e* as internal controls was significantly affected by cold storage. The transcripts of *C. pallens ILP*s were dramatically less abundant in females emerging from cold-stored cocoons than in those emerging from unstored cocoons. Among the three *ILP*s, *CpILP1* showed maximum reduction in mRNA level in females from the cold-stored group (Fig. 6).

## Discussion

To date, different methods, including diapause induction and cold storage have been tried to increase availability and flexibility in commercial production of natural enemies^38,39^. Diapause is a programmed arrest of development that is controlled by endogenous physiological factors. Once induced, the diapause state cannot be terminated by favorable environmental conditions until the developmental process is completed^40–43^. For example, when prepupae diapause was triggered in the green lacewing *C. pallens*, the prepupae stage was extended from 6.4 days to 49–82 days, depending on photoperiod and tempterature^39^. Thus, the extension of developmental stage becomes a great impairment to the flexibility and efficiency in mass production. Unlike diapause, cold storage is just a state of dormancy known as quiescence, which is directly imposed on the organisms by unfavorable and acute change in environmental conditions, such as low temperature^43^. Typically, development will be immediately restored once the adverse conditions come to an end^22,44^. In recent decades, the effects of cold storage on the performance of parasitoids have received considerable attention, but that of predators did not. The green lacewings, *C. pallens* is one of the most important naturally occurring predators and rank as some of the most commonly applied natural enemies^44^. To our knowledge, there are no reports concerning the chilling treatment of this species to data. So, it is very meaningful to evaluate the effects of cold storage on this predatory species. Now, tolerance to cold has been analyzed in various natural enemy insects under different temperatures from 4 to 13 °C^11,17,22,33,38^. In view of the serious detrimental effects triggered by low temperatures below 10 °C, we adopted 10 °C in present cold storage regime. Maintaining *C. pallens* cocoon at 10 °C for 20 days did not seriously damage the performance of the emerged adults, suggesting that pupal stage is suitable for short-term cold storage of this species.

It is noteworthy that the preemergence period of *C. pallens* adults was gradually shortened by the extension of storage duration. After being stored for 60 days, the period from the end of cold storage to emergence was just 47.5% of that in unstored control (Table 2). This led us to conclude that the pupae still underwent development during storage at 10 °C. As the variation in development period is indicative of the fluctuation of development rate, it is usually served as a basis for determining the temperature threshold for development. For instance, in the parasitoid *M. raptor*, the developmental period after return to 25 °C showed no obvious changes after storage at 10 °C, but was significantly shortened after storage at 13 °C. So, the lower temperature threshold for development of *M. raptor* was estimated between 10 °C and 13 °C^11^. In a mysarid wasp *G. ashmeadi*, the overall development time after removal from cold storage at an average temperature of 6 °C was significantly extended, when compared to unstored control^22^. The lower threshold of development of *G. ashmeadi* was estimated at 8.5 °C, above 6 °C^45^. By this logic, the low temperature threshold for pupae development in *C. pallens* should be below 10 °C, rather than above 10 °C.

Decline of fecundity is very common in cold-stored insects, especially in parasitoids^17^. The damage to the reproductive system caused by chilling conditions was viewed as the reason^19,46,47^. By measuring digestive enzyme activities after cold storage, we inferred that recession of digestive ability might be an important inducement for the decline of fecundity in cold-stored *C. pallens*. In lacewings emerging from cold-stored cocoons, lipase activity was significantly lower than that in unstored controls, suggesting a reduction in fat catabolism. As catabolism and anabolism are two opposite processes, a decline in fat catabolism can theoretically lead to a promotion of fat reserves as long as fat anabolism is not weakened. Similarly, the decrease in protease and trehalase activities not only reflected a drop in protein and glucose catabolism, but also indirectly suggested an increase in reserve of these two nutrients. So, the metabolic arrest in *C. pallens* females in cold-stored group is caused by catabolic suppressions of above three macronutrients.

So far, insulin signaling pathway has been implied in different physiological process such as immune response, development, longevity, metabolism and reproduction^48,49^. Our prior study also implicated that insulin-like peptides (ILPs) are crucial upstream players in nutritional and reproductive signaling^35^. Here, we confirmed once more the pivotal roles of *C. pallens ILPs* in reproductive regulation by demonstrating that application of bovine insulin restored lacewing fecundity after cold storage. Presently, the etiology of chilling injury in insects is still relatively unknown. Gathering of toxic metabolites and reduction of energy reserves were implied as possible reasons^50^. Insect ILPs are a kind of endocrine hormone secreted by media neurosecretory cells (MNCs)^51^. In present study, transcripts level of *C. pallens ILP*s in females emerging from cold-stored cocoons was significantly lowered (Fig. 6). This led us to presume that irreversible damage to the MNCs caused by low temperature may be the reason for chilling injury characterized by reproductive decline in *C. pallens*. The logic is that the harm to the nerve cells reduced insulin secretion, impaired insulin signaling, inhibited ovarian development and ultimately decreased reproductive output. The green lacewing *C. pallens* has three *ILP*s^35^. Quantitative PCR analysis revealed that the transcripts abundance of *CpILP1* in adult lacewings emerging from cold-stored cocoon was about 49% of that in adult lacewings emerging from unstored controls. *CpILP2* and *CpILP3* showed similar expression patterns with *CpILP1*. Regretfully, we are still not sure whether all of the three *ILP*s participate in lacewing reproductive regulation. Further work is needed to elucidate this query.

The total fecundity of females emerging from cold-stored cocoons was only recovered, in part, by topical application of bovine insulin and apparent difference in egg amount was still present between thecold-stored and unstored groups (Fig. 5). We inferred that this is because bovine insulin is not as efficient as ILPs in insect body^51^. The green lacewing *C. pallens* has attractive application prospect in management of various insect pests. Here, we demonstrated ILPs have the potential to be used as reproductive stimuli in insect after cold storage. The data will contribute to the commercial production of this predatory species.
